# Multi-objective optimization can balance trade-offs among boreal caribou, biodiversity, and climate change objectives when conservation hotspots do not overlap

**DOI:** 10.1038/s41598-022-15274-8

**Published:** 2022-07-13

**Authors:** Amanda E. Martin, Erin Neave, Patrick Kirby, C. Ronnie Drever, Cheryl A. Johnson

**Affiliations:** 1 Environment and Climate Change Canada, Science and Technology, National Wildlife Research Centre, Ottawa, ON K1S 5B6 Canada; 2 Department of Biology, Carleton University, Ottawa, ON K1S 5B6 Canada; 3 Nature United, Ottawa, ON Canada; 4 Department of Applied Geomatics, University of Sherbrooke, Sherbrooke, QC J1K 2R1 Canada

**Keywords:** Boreal ecology, Conservation biology

## Abstract

The biodiversity and climate change crises have led countries—including Canada—to commit to protect more land and inland waters and to stabilize greenhouse gas concentrations. Canada is also obligated to recover populations of at-risk species, including boreal caribou. Canada has the opportunity to expand its protected areas network to protect hotspots of high value for biodiversity and climate mitigation. However, co-occurrence of hotspots is rare. Here we ask: is it possible to expand the network to simultaneously protect areas important for boreal caribou, other species at risk, climate refugia, and carbon stores? We used linear programming to prioritize areas for protection based on these conservation objectives, and assessed how prioritization for multiple, competing objectives affected the outcome for each individual objective. Our multi-objective approach produced reasonably strong representation of value across objectives. Although trade-offs were required, the multi-objective outcome was almost always better than when we ignored one objective to maximize value for another, highlighting the risk of assuming that a plan based on one objective will also result in strong outcomes for others. Multi-objective optimization approaches could be used to plan for protected areas networks that address biodiversity and climate change objectives, even when hotspots do not co-occur.

## Introduction

The earth is facing twin crises of climate change and biodiversity loss. The Intergovernmental Panel on Climate Change (IPCC) has affirmed that the climate is changing at a rate not seen for at least 2000 years^1^. In addition to a global increase in average surface temperatures, we are experiencing increases in extreme weather events—including flooding, droughts, heat waves, and tropical cyclones—that threaten both humans and wildlife species^1^. It is estimated that the rate and magnitude of species loss are similar to—or even exceed—those experienced during the last five mass extinction events^2,3^. And the extinction rate is accelerating, with human-caused habitat loss and deterioration as the primary threats to species^4^. In fact, the Intergovernmental Science-Policy Platform on Biodiversity and Ecosystem Services estimates that more than 500,000 terrestrial species do not have enough habitat to support long-term persistence^4^.

The threats posed by climate change and biodiversity loss have led to international calls for action. The United Nations (UN) Framework Convention on Climate Change came into force in 1994, with the ultimate goal of stabilizing greenhouse gas (GHG) concentrations and preventing dangerous rates of climate change^5^. One hundred and ninety-six Parties have signed on to the 2015 Paris Agreement, which operationalizes commitments made under the Convention, including specific emission-reduction targets for signatories^6^. Conserving biodiversity is one of the main objectives of the UN Convention on Biological Diversity, which was adopted in 1993^7^. In 2010, the 150 signatories of this Convention endorsed a plan to halt global biodiversity loss, including a target to protect at least 17% of terrestrial and inland waters by 2020 (Aichi Target 11)^8^.

Canada’s actions to address its commitments under the UN Conventions on Climate Change and Biological Diversity could play a significant role in efforts to address climate change and biodiversity loss. Canada’s potential to reduce global GHG in the atmosphere is, at least in part, due to its large expanse of boreal forest. Boreal and tropical forests are the largest contributors to the global terrestrial carbon sink, and recent trends (1992–2015) suggest that the contributions of boreal forests are increasing while those of tropical forests decrease^9^. Approximately 5.5 million km^2^ of the boreal forest biome is found in Canada^10^, and it is estimated that this region stores 168–200 trillion kg of carbon in its vegetation, peatlands, and soil^11^. Protection of such existing stores of carbon—and resulting avoidance of GHG emissions from disturbed stores—is a vital component of action to address climate change. Indeed, Drever et al. reported that nature-based climate solutions focused on protection of existing carbon stores (including those in peatlands) had some of the highest potentials for climate mitigation in Canada^12^. This region also contains some of the largest areas of primary forest on Earth and holds more available freshwater than any other single country^10^. It supports billions of birds and significant populations of large carnivores and ungulates that have disappeared from southern portions of their ranges, as well as populations of many range-restricted insects^10^.

In addition to its international commitments, Canada has an obligation to manage and recover populations of species listed under its *Species at Risk Act*^13^. This includes the boreal population of woodland caribou (*Rangifer tarandus*; hereafter “boreal caribou”), which has been listed as Threatened under the *Act* since 2003^14^. Boreal caribou is emblematic of Canada’s boreal region, with a distribution that covers nearly half (46%) of this region (estimated using data from^15,16^). It has been the focus of significant national and international conservation attention, in part because its conservation is at odds with economic development^17–19^. Boreal caribou is a priority population under Canada’s “Pan-Canadian approach to transforming species at risk conservation in Canada”, a multi-species, multi-ecosystem approach to conservation^20^. Priority species/populations are chosen, in part, for their significance to Indigenous Peoples and Canadians^21^. Caribou plays a role in Indigenous culture, traditions, and relationships to the land^22^. Its image has been used on Canadian currency (the 25 cent coin) for almost a century^23^. Priority species/populations are also selected based on the expectation that actions to manage and recover these priorities will provide significant co-benefits for other species at risk and for biodiversity^21^.

Previous research demonstrates that protection of habitat for boreal caribou could help Canada act on its other international and national commitments to address climate change and biodiversity loss^24,25^. Hotspots of high soil carbon storage are found across the boreal caribou distribution, reaching values as high as 3,287 tonnes/hectare^25^. Johnson et al. found that protection of habitat for boreal caribou could benefit 80 other at-risk species that require management and recovery under Canada’s *Species at Risk Act*^25^. Beyond at-risk species, habitat protection could benefit other boreal species; for example, Drever et al. estimated that 90% of Canada’s boreal mammal and bird species are found within the boreal caribou distribution^24^.

Many hotspots of high value for the protection of species at risk, climate refugia, and soil carbon within the boreal caribou distribution are outside of the current protected areas network^25^; thus, there is an opportunity to expand this network to better represent and protect these hotspots. However, there is a catch—areas of high value for representation of species at risk, climate refugia, and soil carbon rarely occur in the same place^25^. For example, locations with high value as climate refugia are not likely to also have large soil carbon stores.

This leads to the question—is it possible to expand the protected areas network in a way that allows for strong representation of areas important for conservation of boreal caribou, other species at risk, climate refugia, and soil carbon stores?

**Figure 1:**
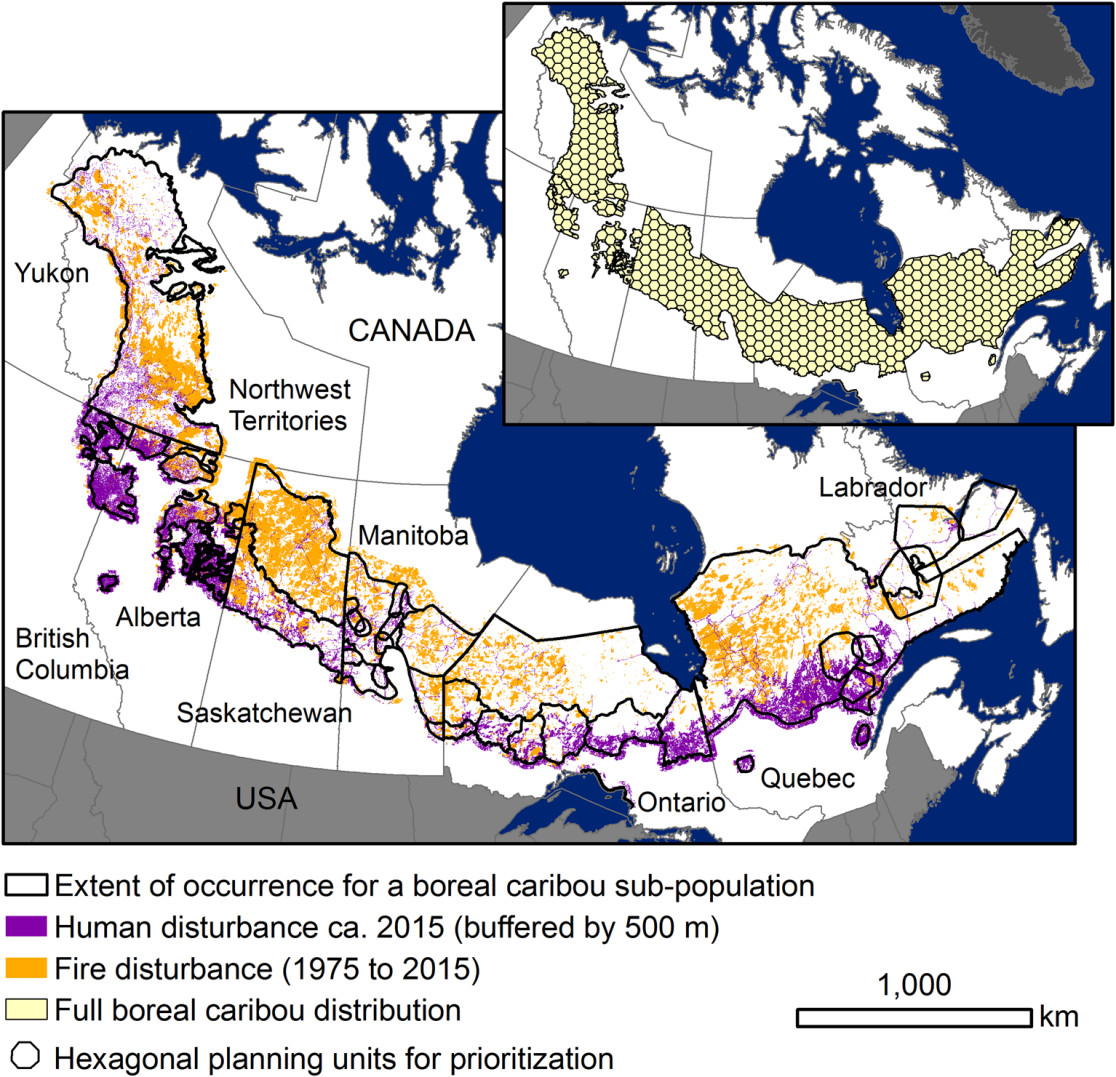
Extent of occurrence for each of 51 boreal caribou sub-populations, as defined in the 2012 Recovery Strategy, and the footprint of human and fire disturbances as of 2015. The inset figure shows the full distribution of boreal caribou, subdivided into 665 planning units using a hexagonal grid. We retained only the portion(s) of each planning unit that overlapped with the boreal caribou distribution, resulting in 316 full, 5000 km^2^ units and 349 partial units with areas of 0.57 to 4999.27 km^2^.

To address our overarching question, we evaluated two alternative planning scenarios. These scenarios had the same six overall conservation objectives—i.e. to expand the protected areas network to maximize the representation of boreal caribou habitat, richness of other (i.e. non-boreal-caribou) species at risk, taxonomic diversity of species at risk, unique species, climate refugia, and soil carbon stores (see Table 1 for full descriptions). Each scenario had the same set of planning units, where a unit is a discrete area that can either be prioritized for addition to the protected areas network or not (see Fig. 1). We also used the same modeling and analysis approach for each scenario. That is, we used linear programming to optimize selection of priority areas to add to the existing protected areas network that address our multiple conservation objectives. We then assessed how close we could get to the best possible outcome for each objective included in the multi-objective plan, and thus how prioritization for multiple, competing conservation objectives could affect the outcome for each individual objective. To identify the best possible outcome for an objective, we used linear programming to select areas to add to the existing protected areas network to maximize representation of value for only that objective.

**Table 1:** Description of data used to calculate the value of each planning unit for six conservation objectives.

Conservation objective	Measure of conservation value for each planning unit	Description of data used to estimate conservation value	Data source
Maximize protection of boreal caribou habitat	Expand Protection scenario: Area of unprotected land and inland waters > 500 m from human disturbance Protect Habitat scenario: Area of unprotected land and inland waters unburned for > 40 years and > 500 m from human disturbance	Footprint of human disturbance within the boreal caribou distribution, manually digitized from 15-m panchromatic imagery	Environment and Climate Change Canada ^63^
		Extent of occurrence of fires occurring between 1975 and 2015	provided to Environment and Climate Change Canada by provinces and territories
Maximize representation of other (i.e. non-boreal-caribou) species at risk	Number of species with extents of occurrence overlapping the unprotected portion of a planning unit	The extent of occurrence for each of 80 species (or other designatable units*) that (a) are listed as Special Concern, Threatened, or Endangered under the *Species at Risk Act*, (b) are found within the boreal caribou distribution, and (c) could benefit from protection of habitat for caribou, i.e. species for which human disturbance has been identified as a threat to persistence. Species were classified into one of nine taxonomic groups (amphibian, arthropod, bird, lichen, mollusc, mammal, reptile, vascular plant, fish)	Johnson et al.^25^
Maximize taxonomic representation of other species at risk	Modified inverse Berger-Parker index = number of species with extents of occurrence overlapping the unprotected portion of a planning unit/number of species in the taxonomic group with the most species		
Maximize representation of unique species	Number of “unique” species with extents of occurrence overlapping the unprotected portion of a planning unit, for the subset of seven species with an extent of occurrence covering ≤ 20% of the boreal caribou distribution and > 50% of their full Canadian extent within the boreal caribou distribution		
Maximize potential as a climate refugia	Sum of refugia potential values for the unprotected portion of a planning unit	Maximum refugia potential for each 1-km^2^ grid cell, where potential = 1 if the cell was projected to remain in the same ecoregion type in 2050, and values decline towards zero as the distance between the ecoregion type in a cell now and the closest cell where that type is projected to be in 2050 (under RCP 8.5) increases	Stralberg et al.^64^
Maximize mass of soil carbon stores	Tonnes of stored carbon within the top 1 m of the soil profile for the unprotected portion of a planning unit	Predicted organic carbon content to a depth of 1 m for each 250 m grid cell; predictions are derived from a set of 158 remote-sensing-based data layers, using machine-learning methods and a set of ~ 150,000 soil profiles for model training	Hengl et al.^65^

Measures of conservation value for each objective were the same in the Expand Protection and Protect Habitat scenarios, with the exception of the objective to maximize protection of boreal caribou habitat.

*Canada’s *Species at Risk Act* includes subspecies, varieties, geographically distinct populations, and genetically distinct populations in its definition of a wildlife species; these are referred to as designatable units (https://cosewic.ca/index.php/en-ca/reports/preparing-status-reports/guidelines-recognizing-designatable-units.html accessed 18/08/2021).

What differed between the two alternative planning scenarios was the primary driver for protection of land and inland waters and our measure of conservation value for boreal caribou. In the “Expand Protection” scenario, the primary driver for protection was Canada’s commitment to expand its protected areas network. Although Canada did not achieve its 2020 target to protect 17% of terrestrial land and inland waters (12.5% protected by 2020^26^), Canada has adopted even more ambitious targets to protect 25% by 2025 and 30% by 2030^27^. In this scenario, we plan to expand the network to protect 30% of the boreal caribou distribution.

In the “Protect Habitat” scenario, the policy driver is Canada’s obligation to protect critical habitat for boreal caribou, i.e. the habitat necessary for the survival or recovery of the population. For boreal caribou, critical habitat is defined as a threshold percent of each sub-population’s range that has not been disturbed by fire for > 40 years and is > 500 m from any human disturbance (e.g. road, mine, harvested forest block)^28,29^. The threshold is 40% for Saskatchewan’s Boreal Shield sub-population and 65% for each of the remaining 50 sub-populations (Fig. 1)^29,30^. In this scenario, we plan to expand the network to meet these “critical habitat targets”. Additionally, recognizing that strict protection may only be part of a larger suite of actions designed to meet critical habitat targets, we also ran analyses using 25, 50, or 75% of each critical habitat target identified above.

The value of each planning unit for boreal caribou was calculated in a slightly different way for our two conservation planning scenarios. In each case, conservation value was informed by the definition of undisturbed habitat in the Recovery Strategy for boreal caribou, i.e. the area of land or inland waters unburned for > 40 years and > 500 m from any human disturbance^29^. For the Expand Protection scenario we calculated the unprotected area of each planning unit that was > 500 m from human disturbance. We did not include fire disturbance because the focus of this scenario was on protected area creation. Generally speaking, management of protected areas puts a greater focus on limiting human disturbance than on disrupting natural disturbance regimes such as fire, although we acknowledge that fire management or control can be implemented as part of the management strategy for some protected areas. Thus, we expect that designation of a protected area would be more likely to reduce human disturbances (e.g. resource extraction activities) in that area than to limit fire. In fact, a previous study suggests that fires may be more likely in protected areas in North America than outside of them^31^. For the Protect Habitat scenario, we estimated conservation value using the full definition of undisturbed habitat.

## Results

### Expand Protection: can areas selected to meet Canada’s commitment to expand its protected areas network also capture other key conservation values?

**Figure 2:**
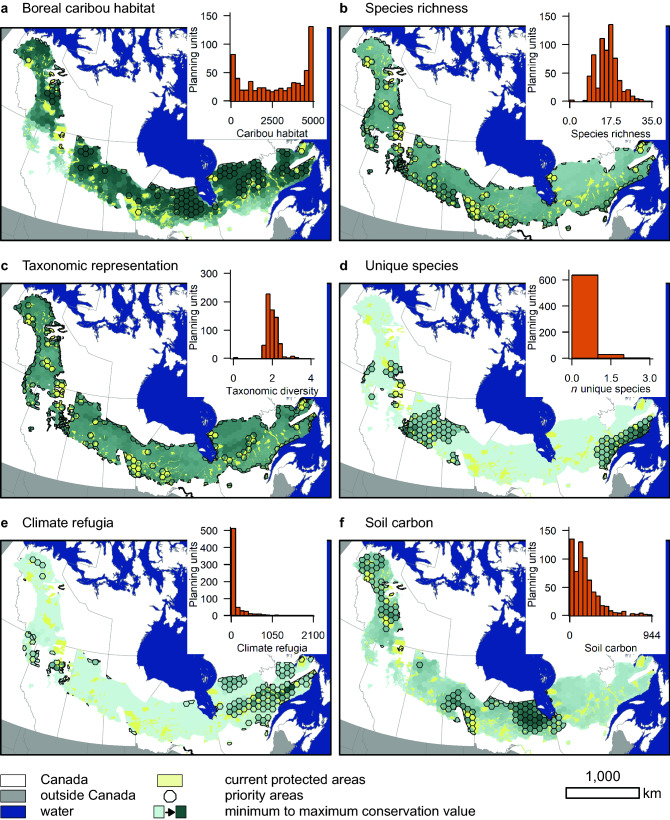
Areas prioritized when adding 19.5% of the boreal caribou distribution to the protected areas network rarely aligned for the six conservation objectives, i.e. to maximize (**a**) boreal caribou habitat, (**b**) representation of other (i.e. non-boreal-caribou) species at risk, (**c**) taxonomic representation of other species at risk, (**d**) representation of unique species at risk, (**e**) climate refugia potential, and (**f**) mass of soil carbon. Maps depict the distribution of conservation value across the boreal caribou distribution for each conservation objective and priority areas identified when prioritizing to maximize representation of each individual objective. Inset figures show the distributions of conservation values across the 665 planning units.

We found little overlap among priority areas identified for different individual objectives (Fig. 2, Supplementary Table S1). This is not surprising, given that correlations in conservation values for different objectives were generally weak (Supplementary Table S2). The only clear exception was the large area of overlap between priority areas identified for the species richness and taxonomic representation objectives (Jaccard similarity coefficient *J* = 0.79; Supplementary Table S1).

The best possible outcome (i.e. outcome of prioritization to maximize value for an individual objective) captured between 26% of the maximum possible value (i.e. outcome if all unprotected areas of the boreal caribou distribution were protected) for boreal caribou habitat and 98% for unique species (Supplementary Fig. S1). This reflects differences in the distributions of different conservation values across the boreal caribou distribution. When high values were concentrated in one or a few areas, most were included in the priority set (e.g., Fig. 2d). This was not the case when there were many areas of relatively high value (e.g., Fig. 2a) or when most areas had moderate conservation value (e.g., Fig. 2b).

**Figure 3:**
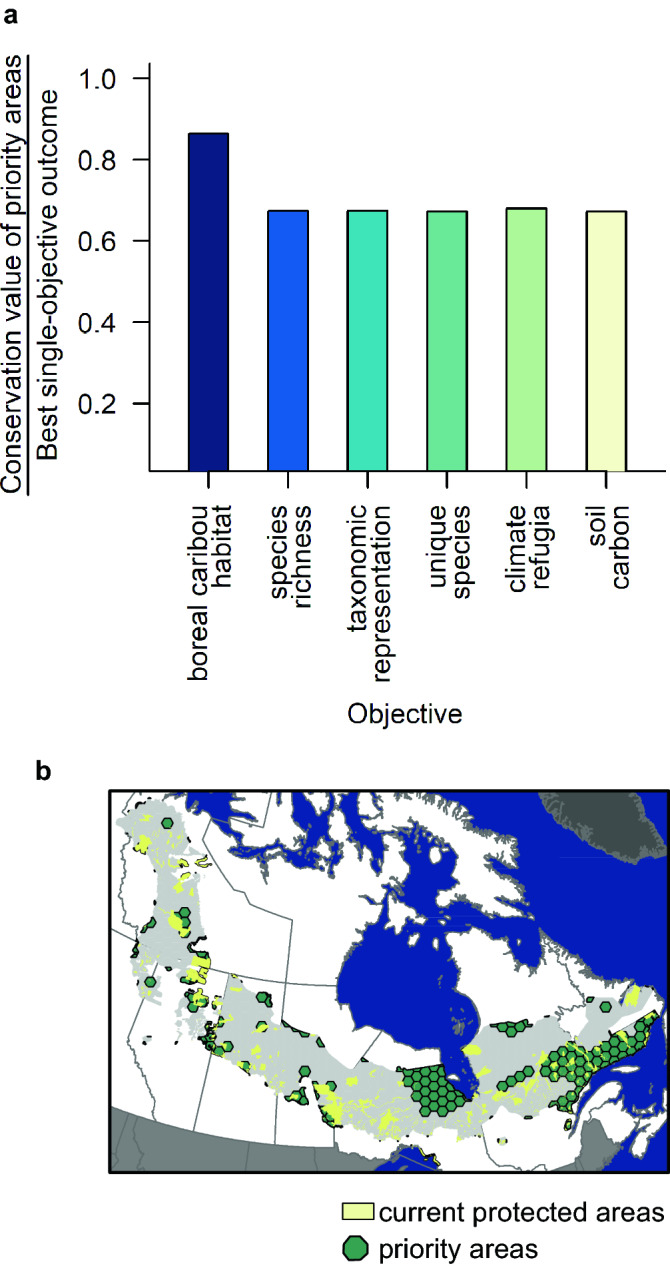
There are opportunities to expand the protected areas network in a way that enhances protection of boreal caribou habitat and areas of high value for other species at risk, climate refugia, and soil carbon stores. (**a**) The conservation value of the priority areas identified using the multi-objective optimization approach was ≥ 0.67 of the best, single-objective outcome for each conservation objective when prioritizing to add 19.5% of terrestrial and inland waters in the Expand Protection scenario. (**b**) Priority areas identified using the multi-objective optimization approach spanned the north–south and east–west extents of the boreal caribou distribution, with the largest priority areas in northern Ontario and southern Quebec.

Despite the small overlap among priority areas identified for individual objectives, we were able to achieve reasonably strong outcomes for all objectives using a multi-objective optimization approach. That is, we achieved at least 67% of the best, single-objective outcome for each conservation objective (Fig. 3a). The best individual outcome in this multi-objective optimization problem was for boreal caribou habitat, achieving 86% of the outcome we got when we focused solely on this as the objective. Prioritized areas spanned the north–south and east–west extents of the boreal caribou distribution, with the largest priority areas in northern Ontario and southern Quebec (Fig. 3b).

**Figure 4:**
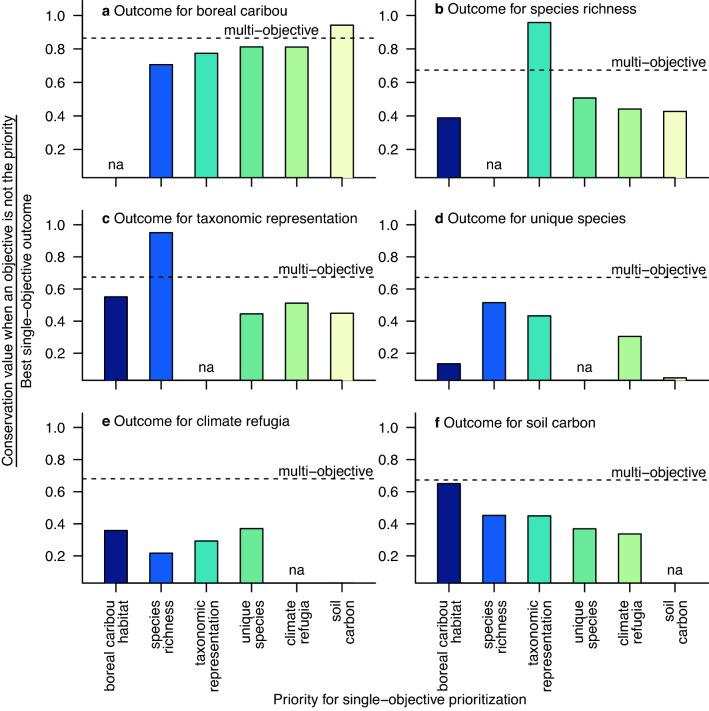
The outcome for a conservation objective was typically worse when we planned to address other, single objectives than when we simultaneously considered all objectives in the Expand Protection scenario. Each figure shows the conservation value of the priority areas as a proportion of the best, single-objective outcome for that conservation objective, comparing the outcome from single-objective prioritizations for each of the other five objectives (bars) to the outcome from the multi-objective approach (dashed line).

The outcome for a conservation objective was typically worse when we planned to address other, single objectives than when we simultaneously considered all objectives. In fact, the outcome was always better for an objective in the multi-objective case than when we prioritized to address other conservation objectives, with the following three exceptions (Fig. 4). Outcomes were better for boreal caribou habitat when prioritizing for soil carbon stores, for species richness when prioritizing for taxonomic representation, and for taxonomic representation when prioritizing for species richness (Fig. 4a–c).

### Protect Habitat: can areas selected to meet Canada’s obligation to protect critical habitat for boreal caribou also capture other key conservation values?

Eighty-nine percent of unprotected land and inland waters within the boreal caribou distribution had to be prioritized for protection when we planned to expand the network to meet the critical habitat targets (Figs. S2, S3). In contrast, only 16% had to be prioritized for protection when we set targets for protection to 25% of each critical habitat target.

**Figure 5:**
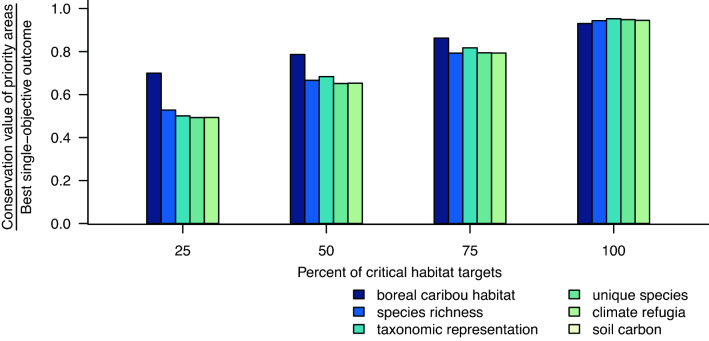
The ability to simultaneously achieve strong outcomes for our conservation objectives depended on how much caribou habitat we aimed to protect for each of 51 sub-populations in the Protect Habitat scenario. We used a critical habitat target of 40% of the Boreal Shield sub-population’s range in undisturbed habitat and a target of 65% for each of the remaining 50 sub-populations, and then ran additional analyses at 25, 50, and 75% of each critical habitat target. The figure shows the conservation value of the priority areas as a proportion of the best, single-objective outcome for that conservation objective. Multi-objective optimization was able to achieve ≥ 0.93 of the best, single-objective outcome for each conservation objective when using the critical habitat targets, but ≥ 0.49 when using 25% of each target.

The ability to prioritize areas that simultaneously achieve high value for all conservation objectives depended on the amount of caribou habitat we aimed to protect for each sub-population. The conservation value of the priority areas ranged from ≥ 93% of the best possible (i.e. single-objective) outcome for each conservation objective when we planned to expand the network to meet the critical habitat targets, to ≥ 49% when we set targets for protection to 25% of each critical habitat target (Fig. 5).

**Figure 6:**
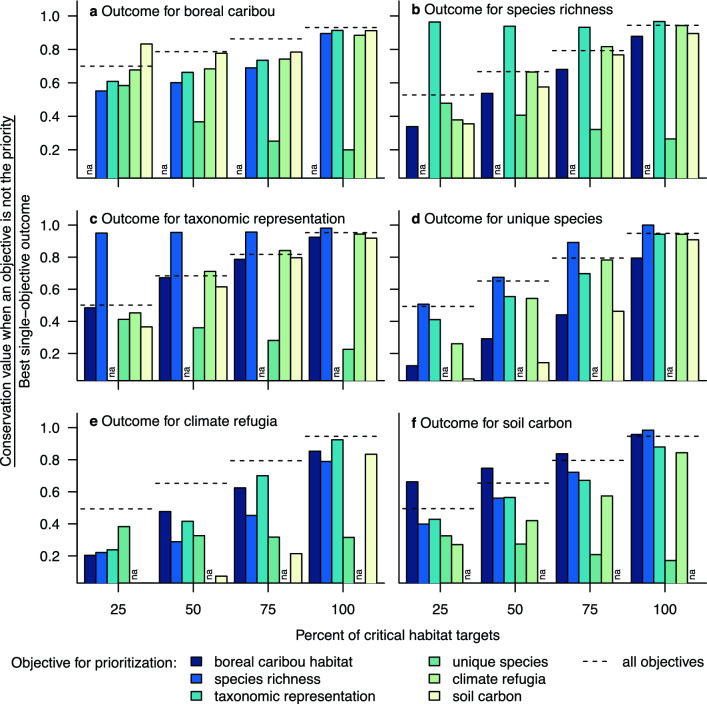
The outcome for a conservation objective was typically worse when we planned to address other, single objectives than when we simultaneously considered all objectives in the Protect Habitat scenario. Each figure shows the conservation value of the priority areas as a proportion of the best, single-objective outcome for that conservation objective, comparing the outcome from single-objective prioritizations for each of the other five objectives (bars) to the outcome from the multi-objective approach (dashed line). We used a critical habitat target of 40% of the Boreal Shield sub-population’s range in undisturbed habitat and a target of 65% for each of the remaining 50 sub-populations, and then ran additional analyses at 25, 50, and 75% of each critical habitat target.

Nevertheless, the outcome for a conservation objective was usually worse when we planned to address other, single objectives than when we simultaneously considered all objectives, even at the lowest targets for protection (Fig. 6). The benefit of explicitly prioritizing for multiple conservation objectives when planning for protection of boreal caribou habitat was particularly apparent for climate refugia and soil carbon (Fig. 6e–f).

## Discussion

Our results suggest we cannot expect easy “win wins” when planning for protection of boreal caribou habitat, other species at risk, climate refugia, and soil carbon stores within an expanded protected areas network. We found little overlap among areas prioritized to maximize value for individual objectives. This result is not surprising, given that Johnson et al. also found few areas of overlap among hotspots of high conservation value for species at risk, climate refugia, and soil carbon within the boreal caribou distribution^25^. This finding is also not unique to our conservation objectives or study region. For example, Mitchell et al.^32^ estimated that, while hotspots for carbon storage, freshwater, and nature-based recreation services covered 48% of Canada’s land base, areas identified as hotspots for all three service types were extremely rare, limited to 0.6% of the land base. A global-scale analysis also found disconnects between areas important for biodiversity and for climate refugia. Although projected temperature increases were typically greater outside biodiversity hotspots than inside them, the percentage of biodiversity hotspots at a given latitude also classified as climate refugia was ≤ 47% across environments (terrestrial, freshwater, and marine) and climate projection scenarios^33^.

This spatial separation of hotspots of conservation value is cause for concern, given the widespread and urgent need for action to address climate change and biodiversity loss. Climate change is already affecting all areas of the Earth, and the IPCC predicts that surface temperatures will continue to increase until at least 2050 under all GHG emission scenarios^1^. Without strong action to address climate change, average surface temperatures will continue to increase, and with them the frequency and severity of extreme weather events^1^. Simultaneously, the threat of wildlife species extinction is accelerating, as is the loss of essential ecosystem services that wildlife provide^4^. With limited time, money, and other resources for conservation efforts, success depends on our ability to design conservation actions that address multiple climate mitigation and biodiversity objectives.

In particular, there is a pressing need for protected areas planning for climate change mitigation and adaptation. Although implementation of plans to manage protected areas for climate change have historically been rare (e.g.^34^), a growing body of research provides guidance on climate-smart protected areas planning (e.g.^35,36^). Nature-based climate solutions, which focus on reducing atmospheric GHG levels by protecting and expanding natural carbon sinks, could be a promising approach. For example, it is estimated that nature-based climate solutions could cumulatively mitigate ~ 394 Tg of CO_2_ in Canada between 2021 and 2030^12^. Protection of existing carbon stores is an important part of the climate solution for Canada. For example, avoiding peatland disturbance—including peatlands found in the boreal region—is estimated to provide the second largest opportunity for nature-based climate mitigation in Canada out of the 24 possible approaches assessed by Drever et al.^12^. Protection of existing soil carbon stores in Canada is of particular importance, both at home and globally, with soil carbon stores making up an estimated 96% of Canada’s organic carbon stocks and 20% of the world’s soil carbon stores^37^.

Our findings demonstrate that it is possible to make strategic decisions when expanding Canada’s protected areas network that would address climate change and biodiversity objectives, even when there is little overlap among hotspots of high value for the different objectives. Optimization methods such as the one we use here can facilitate strategic decision-making when planning involves many, in some cases conflicting, objectives. Research suggests that we can expect “better” (e.g. more cost effective) outcomes for conservation plans optimized via linear programming than plans produced using heuristic (rule-of-thumb) approaches^38–40^. However, there are not enough published assessments of systematic conservation planning results—including prioritizations—to confirm this expectation^41^.

Our results also suggest that all conservation objectives should be explicitly considered when planning for expansion of a protected areas network. Although we did not reach the best possible outcome for each objective when we prioritized to expand the network to address multiple objectives, the multi-objective outcome was almost always better than when we ignored one objective to maximize value for another. Previous research by Diaz-Yanez et al. came to a similar conclusion^42^. They found that forest management plans optimized to address multiple objectives (for carbon sequestration, biodiversity, economic profitability of forestry, and timber supply to industry) tended to produce similar or better outcomes across objectives than plans that focused primarily (or exclusively) on a single objective^42^. Such findings have an important implication for conservation planning: that conservation plans based on the assumption that addressing one objective will also result in strong outcomes for others can produce poorer outcomes than plans where all objectives are considered at the outset of planning. This adds to the growing body of literature showing the benefits of designing conservation actions to address multiple conservation and societal goals, and resulting calls to adopt approaches to planning that focus on more than the area-based targets for protection^43,44^.

Returning to our initial question: is it possible to expand the protected areas network in a way that allows for strong representation of areas important for conservation of boreal caribou, other species at risk, climate refugia, and soil carbon stores? Our results suggest the answer is most likely “yes”. However, application of our optimization approach to real-world protected areas planning would require more careful consideration of which areas could be protected. For this exercise, we made the simplifying assumption that all currently unprotected areas within the boreal caribou distribution could be protected through creation or expansion of protected areas, or through other effective area-based conservation measures. In practice, if prioritized areas are not available for protection, then the optimized plan cannot be fully implemented.

Additionally, there is a need to respect the rights of, and to engage with, Indigenous Peoples and local communities when planning where to expand protected areas. Indigenous involvement in protected areas designation and management, including through the development of Indigenous Protected and Conserved Areas, has the potential to support the ongoing process to build and maintain respectful relationships (reconciliation), and to embrace Indigenous ecological knowledge and approaches to conservation and resource management^45,46^. Such approaches have the potential to benefit both people and nature; for example, Schuster et al. found that vertebrate biodiversity on Indigenous-managed lands was similar to that found in protected areas, including in Canada^47^.

Planning should consider the time frame that will be used to monitor and evaluate conservation success, because this can affect how conservation value is measured. For example, our measure of conservation value for boreal caribou habitat focused on areas that are currently undisturbed by humans/fire, rather than areas of high potential for habitat restoration or regeneration. Protection of undisturbed areas is vital for boreal caribou, given the decades it takes for regeneration of boreal caribou habitat^48,49^. However, a longer-term perspective on population management and recovery may require placing additional value on disturbed areas that could be restored.

Planning should also, where possible, include consideration of variations in the price to acquire different areas of land/water for protection or cost to implement other area-based conservation measures in different places. Here we focused on achieving specific target areas for protection, consistent with Canada’s area-based commitments to expand its protected areas network. However, previous research has shown that using area as a surrogate for costs of land acquisition or stewardship can be inefficient. For example, in their case study Carwardine et al. found that using area as a surrogate for cost made achieving their conservation targets 1.4–2.3 times more expensive than when they directly used land acquisition or stewardship costs^50^.

Another consideration for future conservation planning efforts is the spatial configuration of boreal caribou habitat. For this coarse-scale analysis we focused on prioritizing the amount of land and inland water within a planning unit that was undisturbed, i.e. > 500 m from any human disturbance and, for the Protect Habitat scenario, unburned for > 40 years. Thus, planning units with the same undisturbed area but different configurations of that undisturbed area had equal value within our prioritizations. Our choice to focus on the amounts of undisturbed land/inland water is consistent with the definition of critical habitat for boreal caribou used in Canada’s Recovery Strategy^29^, and research has shown that boreal caribou recruitment rates and adult female survival rates are significantly related to the percent of a sub-population’s range that is undisturbed^30^. These are likely the two most important demographic parameters for woodland caribou (e.g.^30,51^). Nevertheless, the spatial configuration of boreal caribou habitat may influence its quality.

Similarly, connectivity may be another important consideration not directly addressed within the context of our prioritizations. More connected protected areas may have better conservation outcomes than those that are less connected^52,53^, facilitating greater rates of movement among protected areas and reducing rates of movement mortality.

Nevertheless, our findings provide further support for the expectation that actions to conserve and recover boreal caribou could provide conservation co-benefits^21^. Protection of large tracts of undisturbed land and inland waters for boreal caribou could simultaneously protect habitat for many other boreal wildlife species^24,25^. Such protection efforts would also support Canada’s efforts to address climate change, protecting portions of the boreal region’s substantial carbon stores^11^.

Our results also illustrate the value of using multi-objective optimization methods to prioritize for conservation action when decisions are complex and there are many, possibly competing objectives. Here, we focused on how multi-objective optimization could help Canada meet its obligations to manage and recover boreal caribou and other species at risk, and commitments made under the UN Conventions on Climate Change and Biological Diversity. However, the challenges of planning to address multiple conservation objectives are not unique to Canada. Although other countries may have different conservation goals that will ultimately guide how they select areas to meet targets for protecting additional land and waters, there are likely to be cases where win–win areas that simultaneously address all conservation objectives are rare. In such cases, optimization methods could provide a valuable tool to guide planning for a protected areas network that simultaneously addresses the urgent need to limit biodiversity loss and climate change.

## Methods

### Study extent and planning units for prioritization

Our study included the full distribution of boreal caribou. We used the best available data provided by Canadian provincial and territorial governments to Environment and Climate Change Canada (ECCC) ca. 2011^16^ to delineate the boreal caribou distribution, updated to include new information provided to ECCC in 2015 (Fig. 1).

Planning units were defined by intersecting the distribution of boreal caribou with a hexagonal grid. Each hexagonal planning unit was 5000 km^2^, chosen to reflect a reasonable minimum size for a protected area^54^. We retained only the portion(s) of each planning unit that overlapped with the boreal caribou distribution, resulting in 316 full planning units and 349 partial units with areas of 0.6 to 4999.3 km^2^ (Fig. 1).

Each planning unit was further subdivided, separating the protected from unprotected portion of the unit. We considered only the unprotected portion of a unit when calculating its conservation value and the area to be added to the existing protected areas network (see below). We considered an area “protected” if it contributes towards Aichi Target 11^8^; these areas were extracted from the 2019 Canadian Protected and Conserved Areas Database^55^. By our calculations, 10.5% of the boreal caribou distribution was already protected; thus 19.5% more of the boreal caribou distribution would be needed to reach a target of 30%.

### Estimating conservation values for each planning unit

We calculated the value of protecting each planning unit for boreal caribou, other species at risk, climate refugia, and soil carbon from data sets described in Table 1.

Additionally, for the Protect Habitat scenario we calculated the area of undisturbed habitat in the unprotected portion of each planning unit that was within the distribution of each of 51 sub-populations (Fig. 1). We used the extents of occurrence defined in the 2012 Recovery Strategy for boreal caribou^56^ because these are the sub-populations for which habitat recovery objectives were defined under the *Species at Risk Act*^13^.

### Expand Protection: can areas selected to meet Canada’s commitment to expand its protected areas network also capture other key conservation values?

We first estimated the best possible outcome we could get for each conservation objective when selecting at most 19.5% of the boreal caribou distribution for protection. We used linear programming to identify the maximum area of boreal caribou habitat that could be added to the protected areas network. In this problem, each of the 665 planning units could either be selected for protection or not. Each planning unit had a conservation value—the unprotected area > 500 m from human disturbance—and a cost—the area of unprotected terrestrial land and inland waters. See Supplementary Fig. S4 for mathematical formulation of the problem. We then repeated the above analyses for each of the remaining five conservation objectives, with the exception that we used different conservation values for the planning units (see Table 1).

Here, and for subsequent analyses, we used the Jaccard similarity coefficient (also known as the Tanimoto similarity coefficient) to quantify the degree of spatial overlap between priority areas (i.e. planning units selected for protection) from different analyses. We determined whether there was significantly greater/less overlap among priority areas than expected (at α = 0.05) in R^57^ using the ‘jaccard’ package^58,59^.

Next, we developed a multi-objective linear programming problem to simultaneously address all six conservation objectives, formulated as a “maximin” problem. This maximin problem seeks to identify the set of planning units that maximize the minimum summed conservation value of priority areas for the set of conservation objectives, when selecting at most 19.5% of the boreal caribou distribution for protection. We also included an additional set of constraints to address the issue that we set 5000 km^2^ as the appropriate minimum size for a protected area, but 52.5% of planning units were < 5000 km^2^. We addressed this issue by including a “minimum area constraint” for each unit, which allows selection of that unit only if its area plus the protected area of its connecting neighbors was ≥ 5000 km^2^ (Supplementary Fig. S5). We made these constraints elastic, meaning that each constraint could be violated, but a violation would be penalized. See Supplementary Fig. S6 for the mathematical formulation.

Finally, we conducted a sensitivity analysis to determine if the results from our multi-objective optimization scenario depended on the penalties for violations of the minimum area constraints. To do so, we solved our multi-objective problem eight times, each time using a different penalty: 0 (or no penalty), 0.001, 0.01, 0.1, 1, 10, 100, and 1000. The penalty for constraint violation had little effect on the minimum conservation value achieved (Supplementary Fig. S7). The spatial distribution of priority areas did depend on the penalty for constraint violation (e.g. Supplementary Fig. S7a–b); however, there was always significantly more overlap among priority areas than expected (Supplementary Table S3). Therefore, we focus on results from the version with a penalty = 0 in the main text.

We used the SYMPHONY open-source solver^60^ implemented in R^57^ using the ROI^61^ and ROI.plugin.symphony^62^ packages to solve each version of our planning problem, with a 0.5% relative gap tolerance, which allows the solver to return a solution that is very close to the optimal one (i.e. within 0.5% of the value that bounds the best possible solution). If this solution was not found within a 5-h period, we recorded the best feasible solution found within that period.

### Protect Habitat: can areas selected to meet Canada’s obligation to protect critical habitat for boreal caribou also capture other key conservation values?

The problem formulation for the Protect Habitat scenario was the same as the formulation used for the Expand Protection scenario (see above), with three exceptions. First, we included a penalty for expansion of the protected areas network. All else being equal, we would expect the summed minimum conservation value of priority areas to increase with the area prioritized for protection. Thus, this penalty was included to allow exploration of the trade-offs between the conservation outcome and the protected area needed to achieve that outcome (see next paragraph). Second, the constraint on the area that could be added to the protected areas network in the Expand Protection scenario was replaced by 51 constraints that ensure a threshold percent of undisturbed habitat was protected for each boreal caribou sub-population. The target was 40% for Saskatchewan’s Boreal Shield sub-population and 65% for each of the remaining 50 sub-populations (Fig. 1)^29,30^. We note that, for 31 sub-populations there was not enough undisturbed habitat left to meet the target; for these sub-populations, the target was set to the amount of undisturbed habitat. Third, we omitted the objective to maximize conservation value for boreal caribou habitat, because this is already captured by the critical habitat targets. See Supplementary Fig. S8 for the mathematical formulation.

As in the Expand Protection scenario, we estimated the best possible outcome for each conservation objective when prioritizing to meet critical habitat targets. To do this, we first calculated the minimum area needed to satisfy the critical habitat targets for all sub-populations (see Supplementary Fig. S9 for mathematical formulation). We then used this minimum area estimate to calculate the best possible outcome we could get for each individual objective when adding an area equal to that minimum area to the protected areas network, using the same programming problem as we used for the Expand Protection scenario (Supplementary Fig. S4). We repeated this analysis three more times, using 25, 50, and 75% of each critical habitat target.

To solve our multi-objective optimization problem (see Supplementary Fig. S8), we had to specify the penalty for expanding the protected areas network and the penalty for violating a minimum area constraint (i.e. selection of a planning unit where its area plus the protected area of its connecting neighbors was < 5000 km^2^). We used sensitivity analysis to determine the effects of penalties on our multi-objective optimization results, investigating all combinations of eight penalties for expanding the network (0, 0.001, 0.01, 0.1, 1, 10, 100, 1000) × eight penalties for violating a minimum area constraint (0, 0.001, 0.01, 0.1, 1, 10, 100, 1000). We conducted sensitivity analyses using the critical habitat targets and using 25, 50, and 75% of each target, for a total of 256 prioritizations for the Protect Habitat scenario.

The proportion of the boreal caribou distribution that was prioritized for protection was strongly dependent on the penalty for adding terrestrial land and inland waters to the protected areas network (Supplementary Fig. S10), but not the penalty for violating a minimum area constraint (Supplementary Fig. S11). Based on these sensitivity analyses, we report on results from the version with no minimum area penalty and a moderately-high penalty of 10 for adding area to the protected areas network in the main text. See Supplementary Figs. S12–S13 for results from versions with different penalties.

## Supplementary Information

Supplementary Information.

## Data Availability

The data and R code generated and analyzed during the current study are available in the figshare repository, https://doi.org/10.6084/m9.figshare.18991031.
