# Supplementary material for: Multi-objective optimization can balance trade-offs among boreal caribou, biodiversity, and climate change objectives when conservation hotspots do not overlap
Source: Sci Rep. 2022 Jul 13;12:11895. doi: 10.1038/s41598-022-15274-8 (PMC9279314; doi:10.1038/s41598-022-15274-8)
Supplement: Supplementary file 1 — Supplementary Information. [file 41598_2022_15274_MOESM1_ESM.docx]

Multi-objective optimization can balance trade-offs among boreal caribou, biodiversity, and climate change objectives when conservation hotspots do not overlap

Amanda E. Martin, Erin Neave, Patrick Kirby, C. Ronnie Drever, Cheryl A. Johnson

Table S1. Pairwise comparison of the spatial overlap between planning units prioritized to achieve the best possible outcome for each individual conservation objective when selecting at most 19.5% of the boreal caribou distribution for protection. Above diagonal: Jaccard similarity coefficient. Below diagonal: Evaluation to determine whether there was significantly greater (**bold**) or less (*italics*) overlap among priority areas than expected (at α = 0.05). The test statistic is the centered Jaccard similarity coefficient ^1^: a positive value indicates a greater overlap than expected, and a negative value less overlap than expected. P-values (in parentheses) were estimated using 5000 bootstrapped samples.

|  | Boreal caribou habitat | Species richness | Taxonomic representation | Unique species | Climate refugia | Soil carbon |
| --- | --- | --- | --- | --- | --- | --- |
| Boreal caribou habitat |  | 0.18 | 0.22 | 0.06 | 0.19 | 0.23 |
| Species richness | -0.01 (p = 0.63) |  | 0.79 | 0.20 | 0.18 | 0.15 |
| Taxonomic representation | 0.03 (p = 0.06) | **0.50 (p < 0.01)** |  | 0.16 | 0.22 | 0.14 |
| Unique species | *-0.07 (p < 0.01)* | 0.03 (p = 0.06) | 0.00 (p = 0.88) |  | 0.15 | 0.03 |
| Climate refugia | **0.06 (p = 0.01)** | 0.00 (p = 0.98) | **0.04 (p = 0.04)** | 0.03 (p = 0.13) |  | 0.04 |
| Soil carbon | **0.10 (p < 0.01)** | -0.02 (p = 0.25) | -0.03 (p = 0.10) | *-0.09 (p < 0.01)* | *-0.08 (p < 0.01)* |  |

Table S2. Pairwise Kendall’s tau rank correlations in values for the six conservation objectives in the Expand Protection scenario. Conservation values for each objective were calculated for the unprotected portion of each of 665 planning units (see Table 1 for details).

|  | Species richness | Taxonomic representation | Unique species | Climate refugia | Soil carbon |
| --- | --- | --- | --- | --- | --- |
| Boreal caribou habitat | -0.21 | 0.02 | 0.01 | 0.09 | 0.59 |
| Species richness |  | -0.14 | 0.28 | -0.04 | -0.05 |
| Taxonomic representation |  |  | -0.04 | 0.04 | -0.09 |
| Unique species |  |  |  | 0.12 | -0.07 |
| Climate refugia |  |  |  |  | -0.04 |


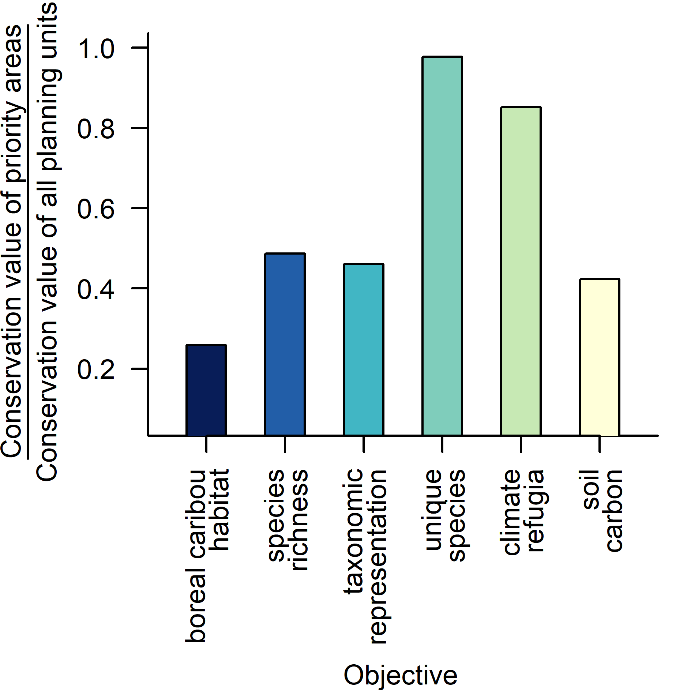


Fig. S1. Best possible outcome for each conservation objective in the Expand Protection scenario, expressed as a proportion of the total possible conservation value, i.e. the summed value for all planning units. Planning units were prioritized using linear programming to maximize the summed conservation value of prioritized units, when selecting at most 19.5% of the boreal caribou distribution for protection.


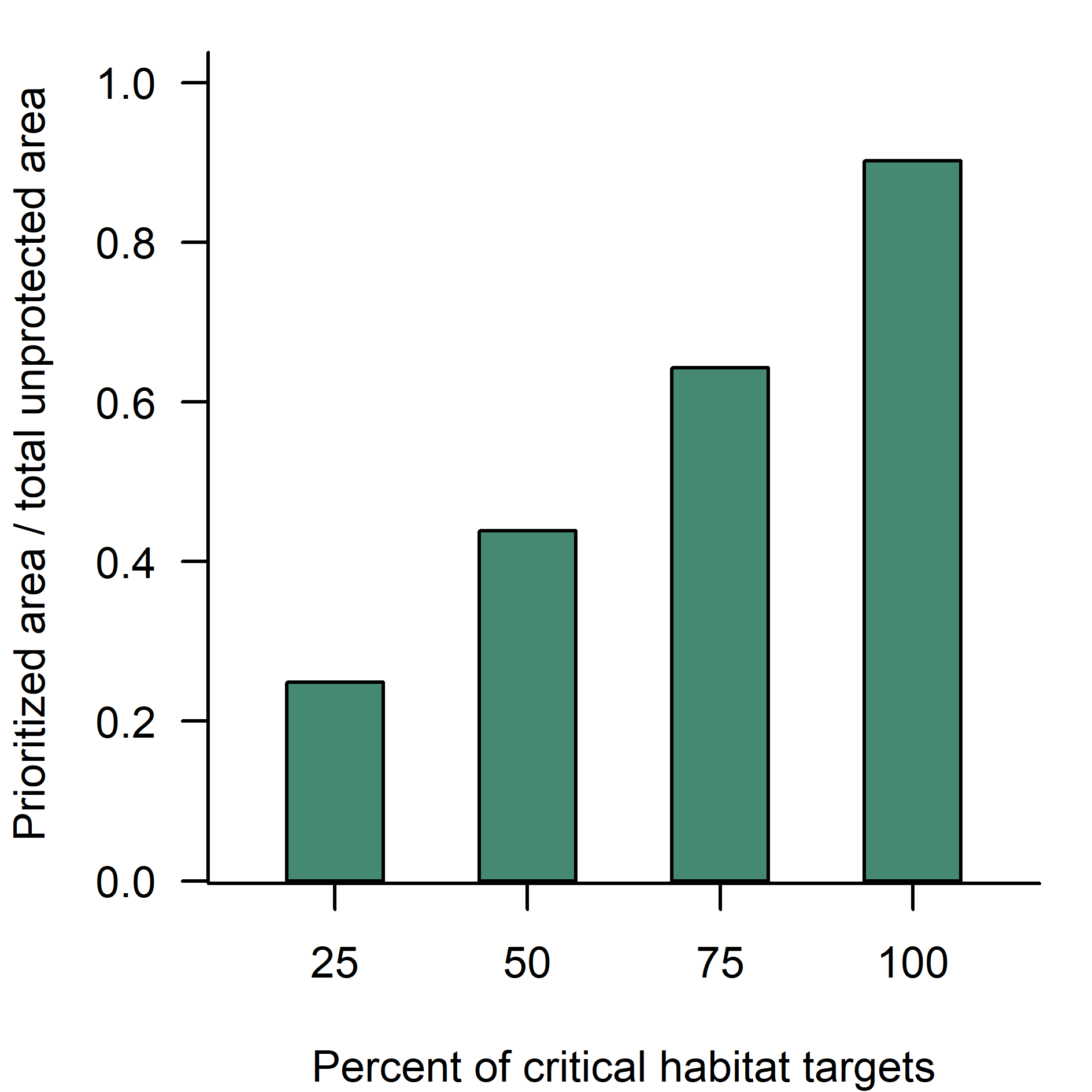


Fig. S2. The proportion of the total unprotected area within the boreal caribou distribution that had to be protected to meet the critical habitat targets (or a percentage of each target) for each of 51 boreal caribou sub-populations in the Protect Habitat scenario. The target was 40% for Saskatchewan’s Boreal Shield sub-population and 65% for each of the remaining 50 sub-populations ^2,3^. Planning units were prioritized using linear programming to minimize the area to be added to the protected areas network, while satisfying the target (or percentage of target) for each sub-population.


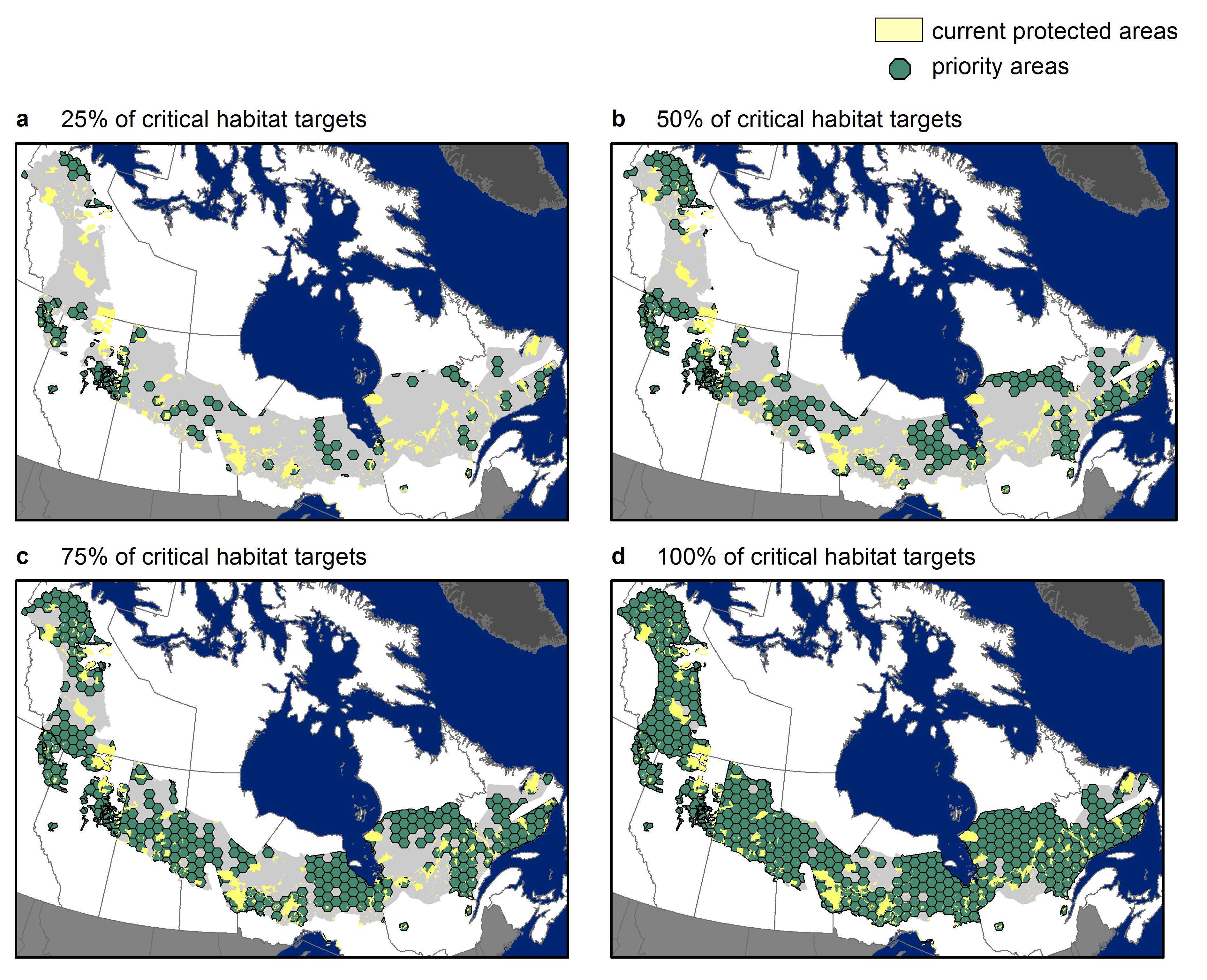


Fig. S3. Priority areas selected to meet the critical habitat targets (or percentage of those targets) for each of 51 sub-populations in the Protect Habitat scenario. The target was 40% for Saskatchewan’s Boreal Shield sub-population and 65% for each of the remaining 50 sub-populations ^2,3^. Planning units were prioritized using linear programming to minimize the area to be added to the protected areas network, while satisfying the critical habitat target (or percentage of target) for each sub-population.


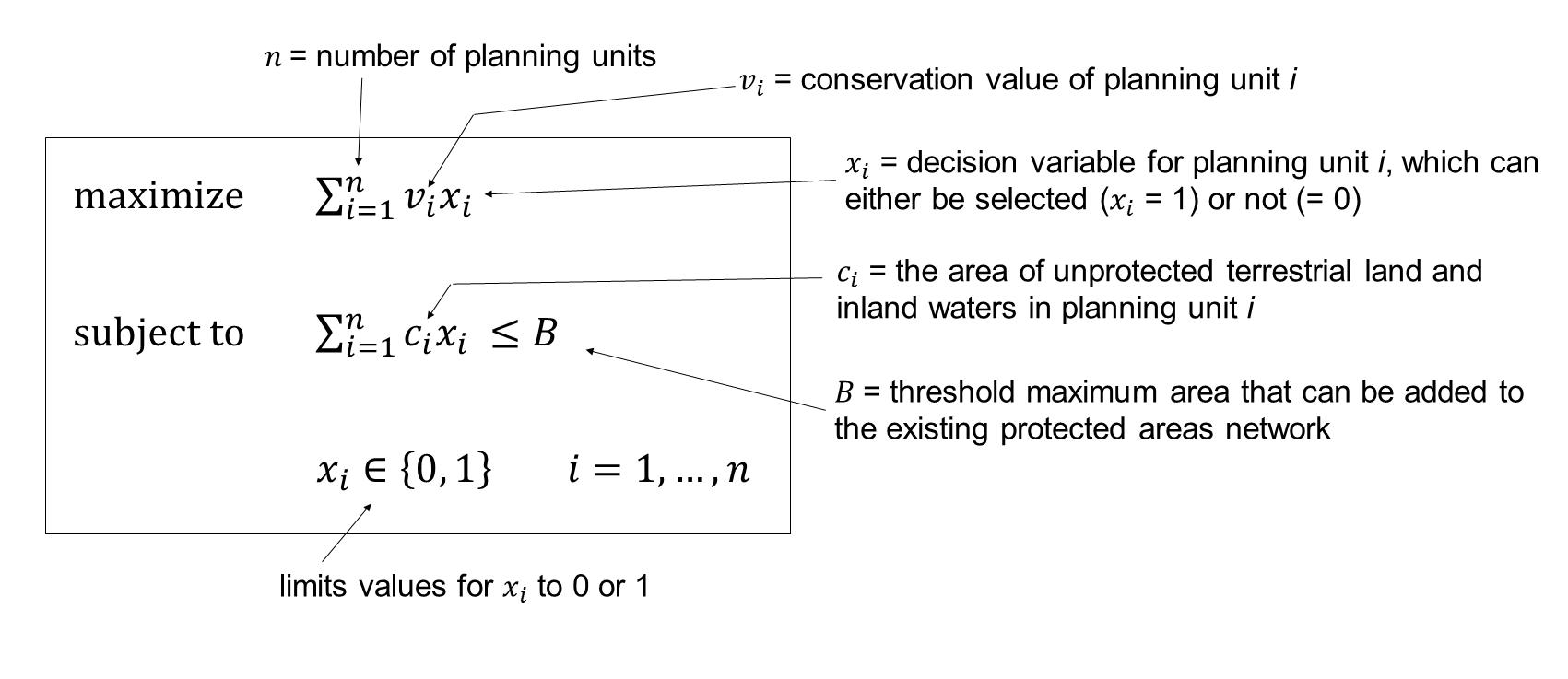


Fig. S4. Annotated mathematical formulation for the Expand Protection single-objective linear programming problem. The objective of this problem is to maximize the summed conservation value of the prioritized planning units, with the constraint that a maximum area equal to 19.5% of the boreal caribou distribution could be prioritized. See Table 1 for description of *v_i_* for each conservation objective.


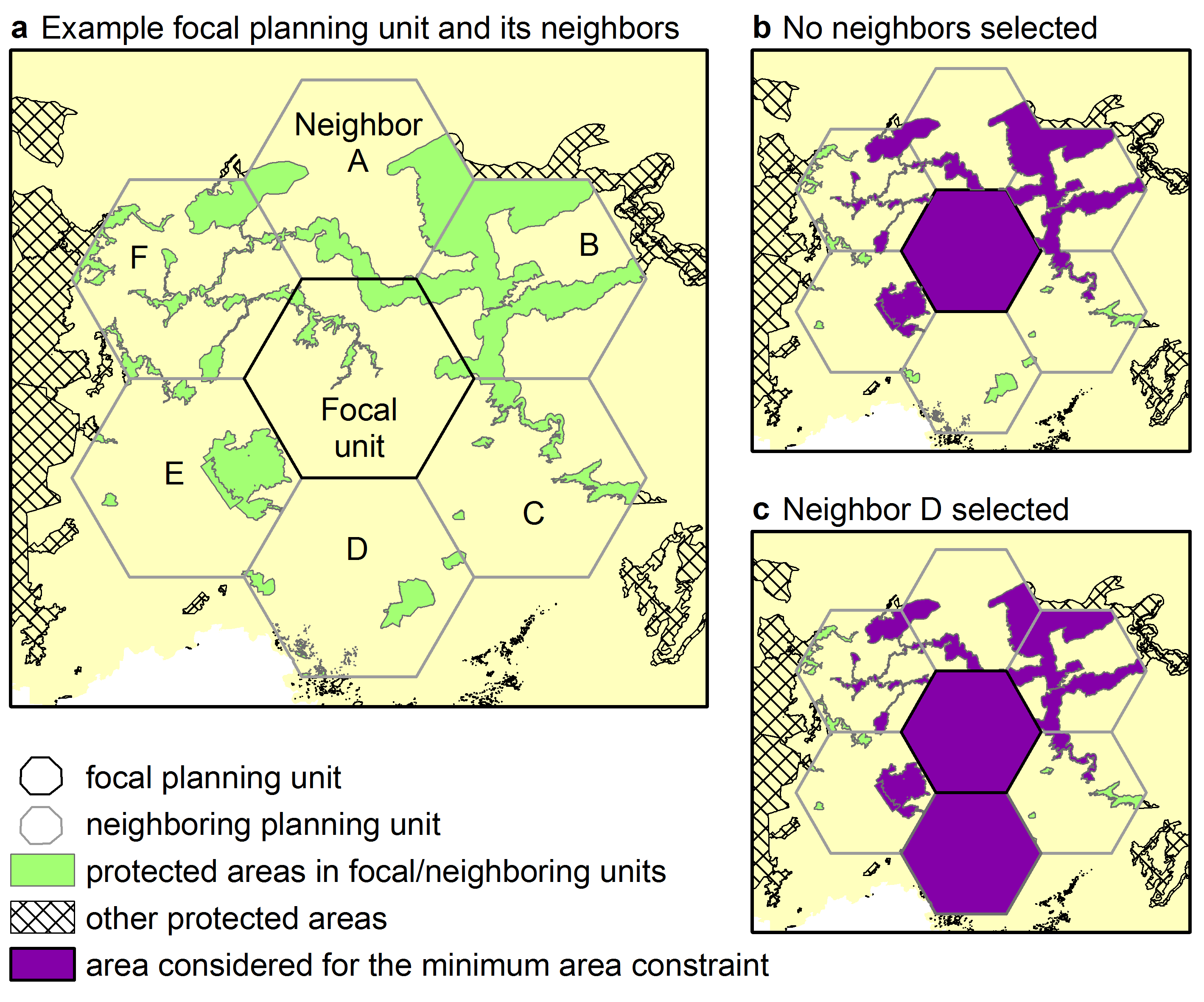


Fig. S5. Illustration of how the area in protection was calculated for the minimum area constraint (*a_ik_* in Figs. S6 and S8, below). A focal planning unit could be selected for protection if its area plus the connected protected area in neighboring planning units was ≥5000 km^2^. If a neighboring planning unit was selected for protection we included the full area of that unit (including currently unprotected and protected portions). However, if that neighboring planning unit was not selected, only the area of existing protected areas (if any) that touched the boundary between the focal and neighboring units was included. For example, if none of the neighboring planning units were selected (b), then we would include the unprotected and protected area in the focal unit plus the protected area in neighboring units that touched the boundary of the focal unit. However, if neighboring unit D was selected for protection (c), we would also include the unprotected area of unit D and the protected area in D that did not touch the boundary of the focal planning unit.


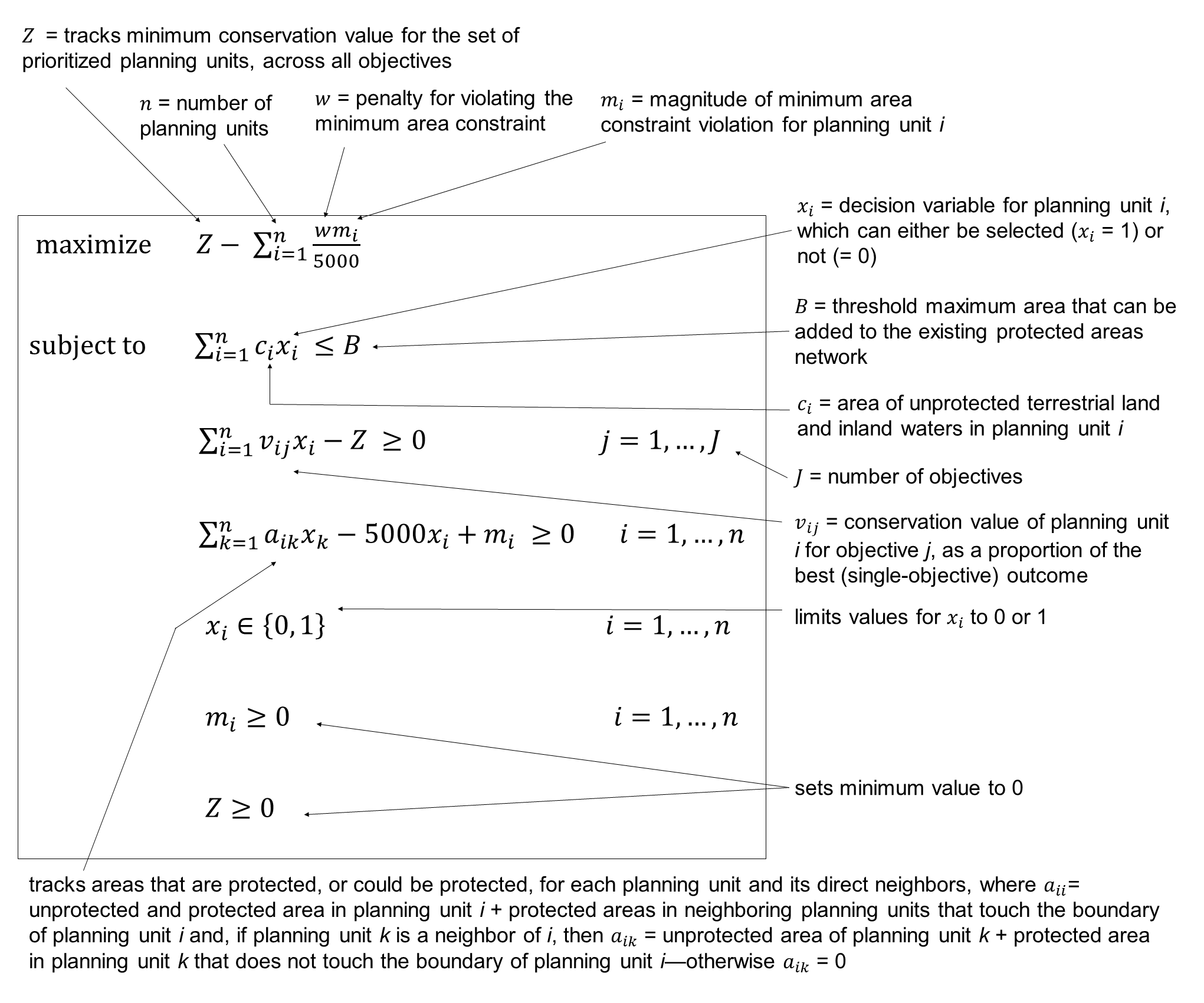


Fig. S6. Annotated mathematical formulation for the Expand Protection multi-objective linear programming problem. The objective of this problem is to maximize the minimum summed conservation value of prioritized planning units, across the set of *J* = 6 conservation objectives. We had a number of constraints for this problem. First, there was a threshold maximum area that could be added to the existing protected areas network of *B* = 19.5% of the boreal caribou distribution. We also had six constraints that allowed for calculation of the minimum conservation value for the set of prioritized planning units (*Z*) across all objectives; see Table 1 for description of values for each of these objectives. Finally, we had a set of 665 constraints to cause selection of a planning unit if its area plus the connected protected area of neighboring units was ≥5000 km^2^. These “minimum area” constraints were elastic, with *m_i_* = amount by which the minimum area constraint was violated for unit *i* (in km^2^). When calculating the objective function value we divided *m_i_* by its corresponding threshold, 5000 km^2^. If, for example, a planning unit is prioritized when its area plus that of connected neighbors is 4500 km^2^, then *m_i_* = 500 km^2^. This violation would reduce the objective function value by 0.0001 when the penalty for constraint violation = 0.001 (0.001 × 500 / 5000) and by 100 when the penalty = 1000 (1000 × 500 / 5000).


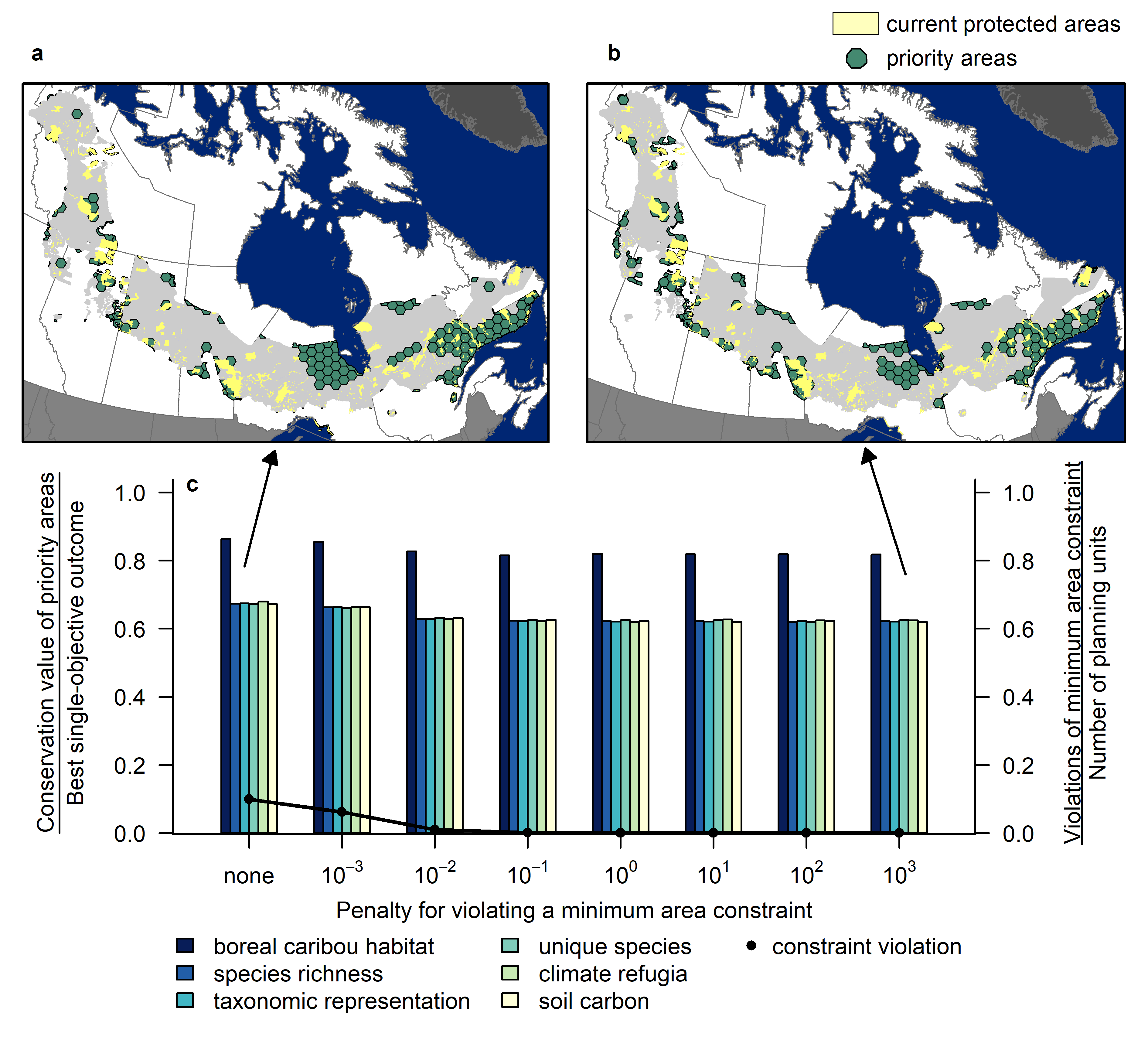


Fig. S7. The penalty for violating a minimum area constraint had little effect on the conservation value achieved for the objectives in the Expand Protection scenario; however, the set of planning units selected did depend on this penalty (see a and b for examples). Panel c shows the conservation value of priority areas as a proportion of the best possible (i.e. single-objective) outcome for that conservation objective, and the number of violations of the minimum area constraint as a proportion of the total number of possible violations (665). Planning units were prioritized using linear programming to maximize the minimum summed conservation value of prioritized planning units, across the set of six conservation objectives, when selecting at most 19.5% of the boreal caribou distribution for protection. The prioritization was repeated eight times, each time using a different penalty for violating a minimum area constraint.

Table S3. Comparison of the spatial overlap between planning units prioritized to simultaneously address multiple conservation objectives in the Expand Protection scenario when there was no penalty for violating a minimum area constraint versus alternatives where penalties varied by six orders of magnitude (from 0.001 to 1000). Comparison are made using the Jaccard similarity coefficient. We evaluated whether there is significantly greater (**bold**) or less (*italics*) overlap among prioritized planning units than expected (at α = 0.05). The test statistic is the centered Jaccard similarity coefficient: a positive value indicates a greater overlap than expected, and a negative value less overlap than expected ^1^. P-values were estimated using 5000 bootstrapped samples.

| Penalty for violating constraint | Jaccard similarity coefficient | Test statistic | P |
| --- | --- | --- | --- |
| 0.001 | **0.74** | **0.56** | **<0.01** |
| 0.01 | **0.57** | **0.39** | **<0.01** |
| 0.1 | **0.56** | **0.38** | **<0.01** |
| 1 | **0.56** | **0.38** | **<0.01** |
| 10 | **0.56** | **0.38** | **<0.01** |
| 100 | **0.55** | **0.37** | **<0.01** |
| 1000 | **0.56** | **0.38** | **<0.01** |


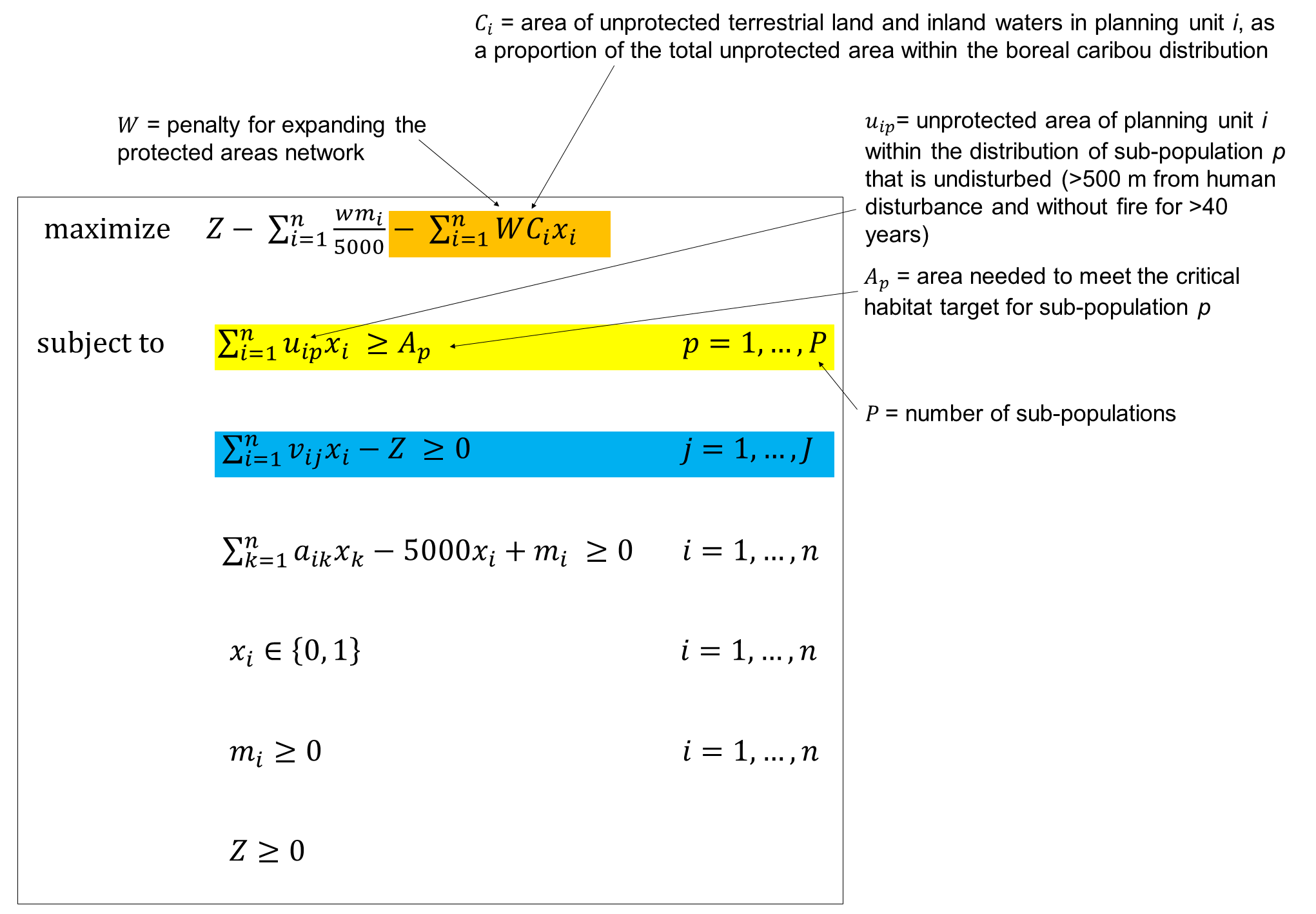


Fig. S8. Annotated mathematical formulation for the Protect Habitat multi-objective linear programming problem. The formulation is the same as the formulation used for the Expand Protection scenario (see Fig. S6, above), with three exceptions. First, there are additional terms included in the objective function (highlighted in orange), to penalize expansion of the protected areas network. Second, the constraint on the priority area in Fig. S6 is replaced by a set of *P* = 51 constraints that ensure a threshold percent of undisturbed habitat is protected for each boreal caribou sub-population (highlighted in yellow). Third, we have five constraints (rather than six) to allow for calculation of the minimum conservation value for the set of prioritized units (*Z*) across objectives (highlighted in blue). We did not include the boreal caribou habitat objective, because the critical habitat targets already capture this.


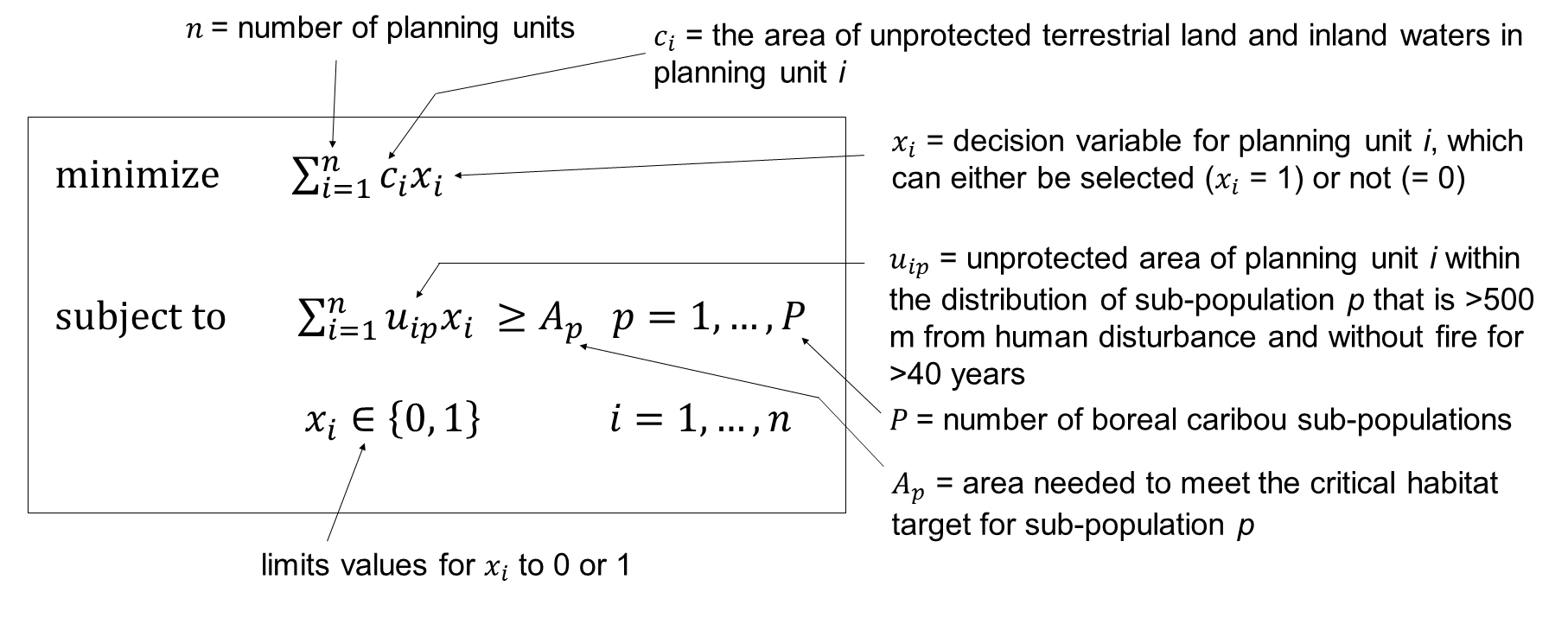


Fig. S9. Annotated mathematical formulation for the Protect Habitat single-objective linear programming problem, used to identify the minimum area that must be added to the protected areas network to meet critical habitat targets (or a percentage of each target). The objective of this problem is to minimize the summed area of unprotected terrestrial land and inland water in the prioritized planning units, while satisfying the *P* = 51 critical habitat targets (or a percentage of each target).


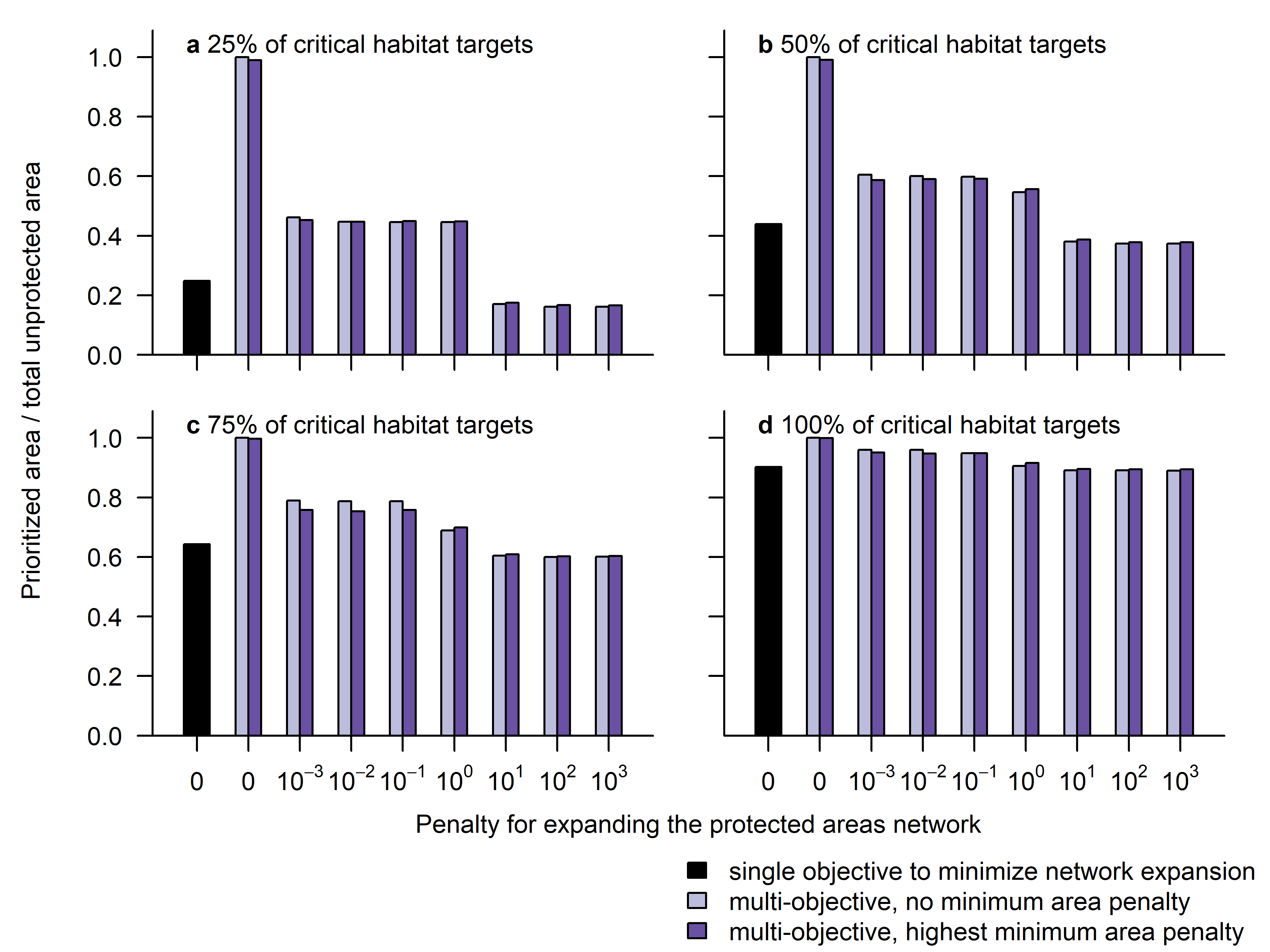


Fig. S10. The proportion of the total unprotected area prioritized for protection in the Protect Habitat scenario depended on the penalty for expanding the protected areas network, particularly when the percent of the critical habitat targets was low (a, b). Each figure depicts the minimum proportion of unprotected area that would need to be protected to satisfy the critical habitat targets (or a percentage of each target) for all sub-populations (from the single-objective optimization; see Fig. S2) and the proportion of undisturbed habitat prioritized when addressing multiple conservation objectives, using a range of penalties for expanding the protected areas network and penalties for violation of a minimum area constraint.


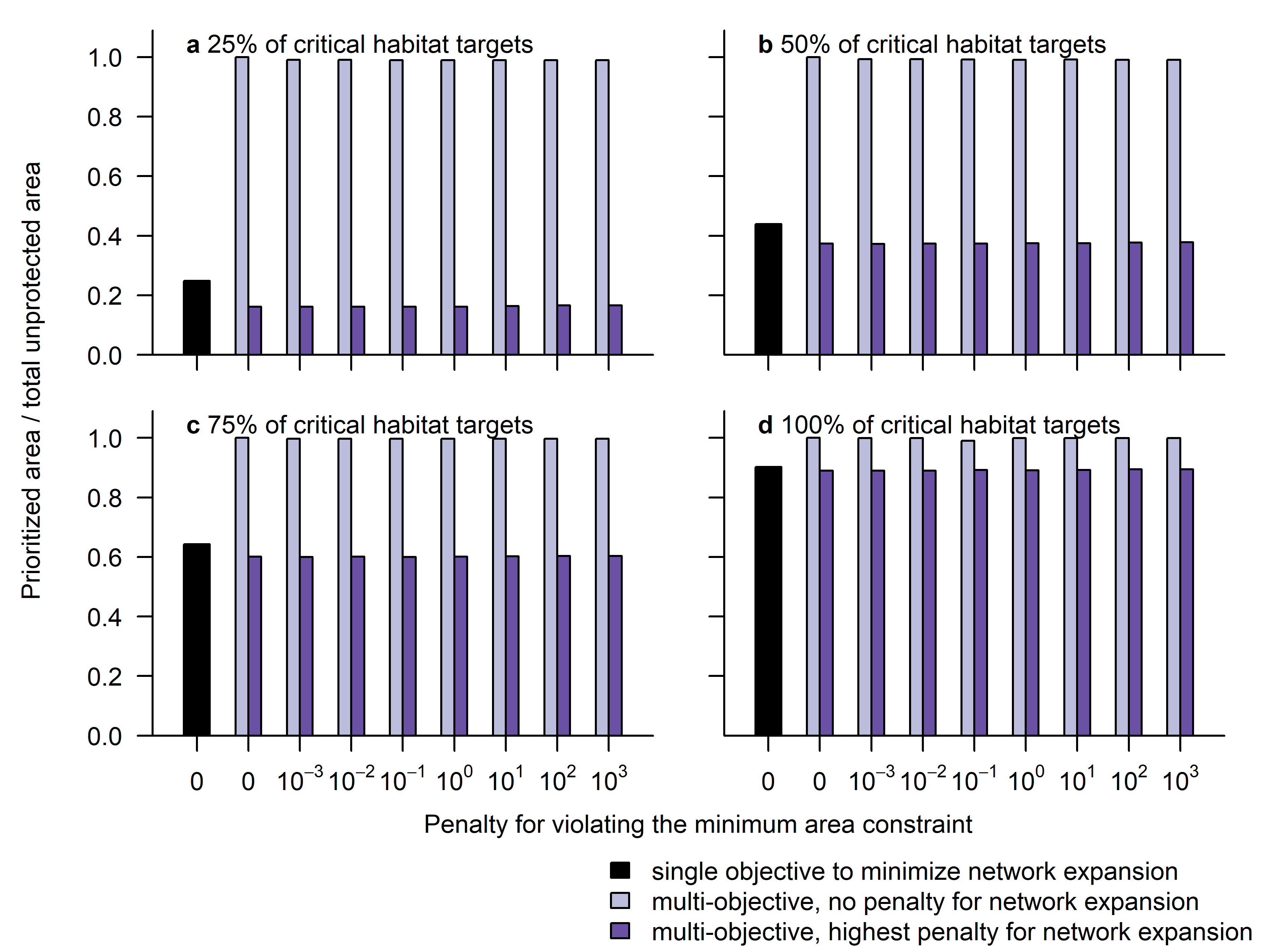


Fig. S11. The proportion of the total unprotected area prioritized for protection in the Protect Habitat scenario was not strongly affected by the strength of the penalty for violating a minimum area constraint. Each figure depicts the minimum proportion of unprotected area that would need to be protected to satisfy the critical habitat targets (or a percentage of each target) for all sub-populations (from the single-objective optimization; see Fig. S2) and the proportion of unprotected area prioritized when addressing multiple conservation objectives, using a range of penalties for expanding the protected areas network and penalties for violation of a minimum area constraint.


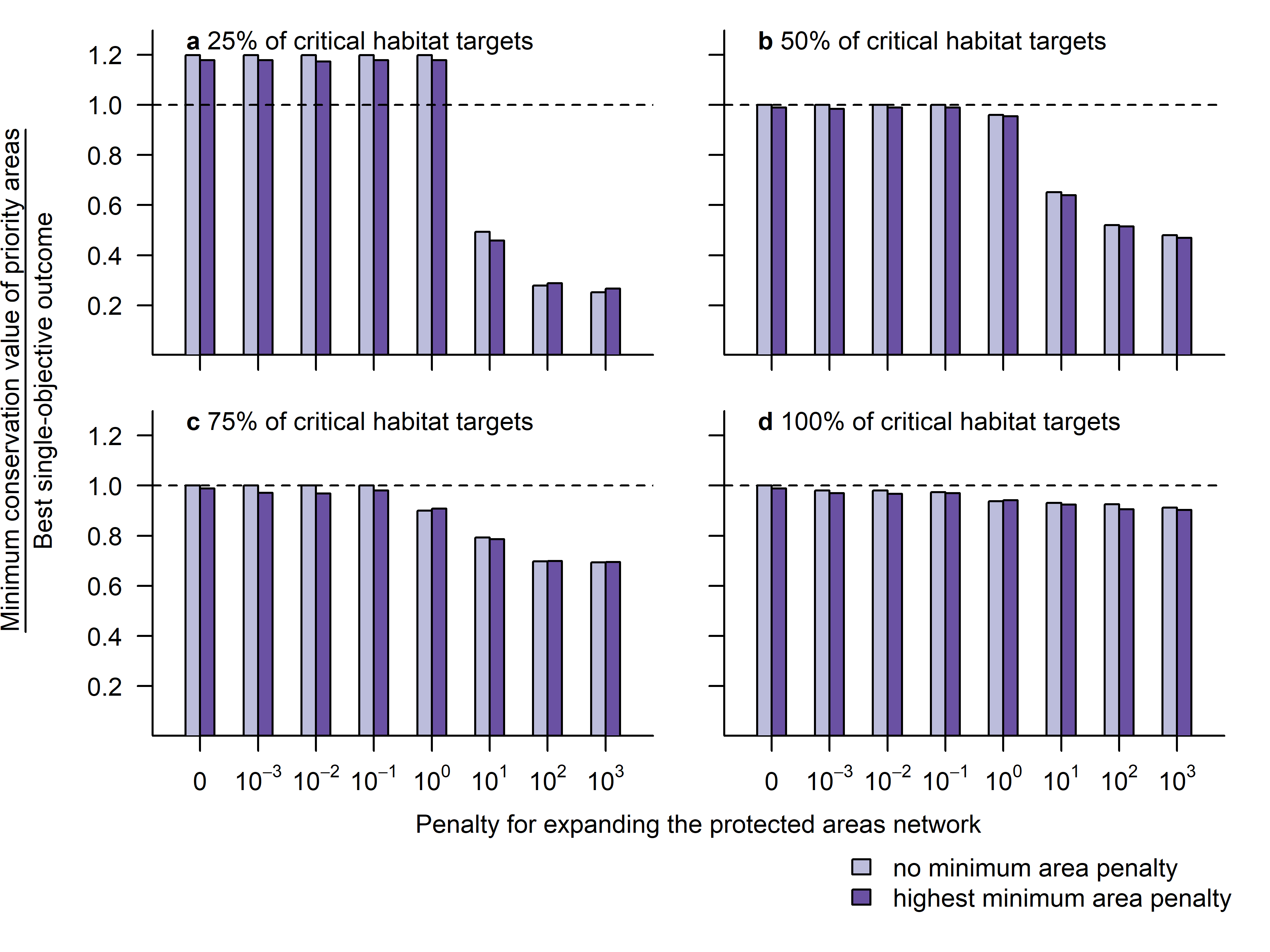


Fig. S12. How close we could get to the best possible outcome when simultaneously prioritizing for all our conservation objectives in the Protect Habitat scenario depended on the penalty for adding terrestrial land and inland waters to the protected areas network. Each figure depicts the minimum conservation value across the set of conservation objectives, as a proportion of the best single-objective outcome (the outcome when prioritizing to maximize each individual objective), for prioritizations using a range of penalties for expanding the protected areas network and penalties for violation of a minimum area constraint. Values >1 indicate that the minimum conservation value achieved across the set of six conservation objectives was higher in the multi-objective prioritization than when each objective was prioritized separately; this was possible when the area prioritized for protection using our multi-objective programming problem exceeded the area prioritized in the single-objective context (see Fig. S10).


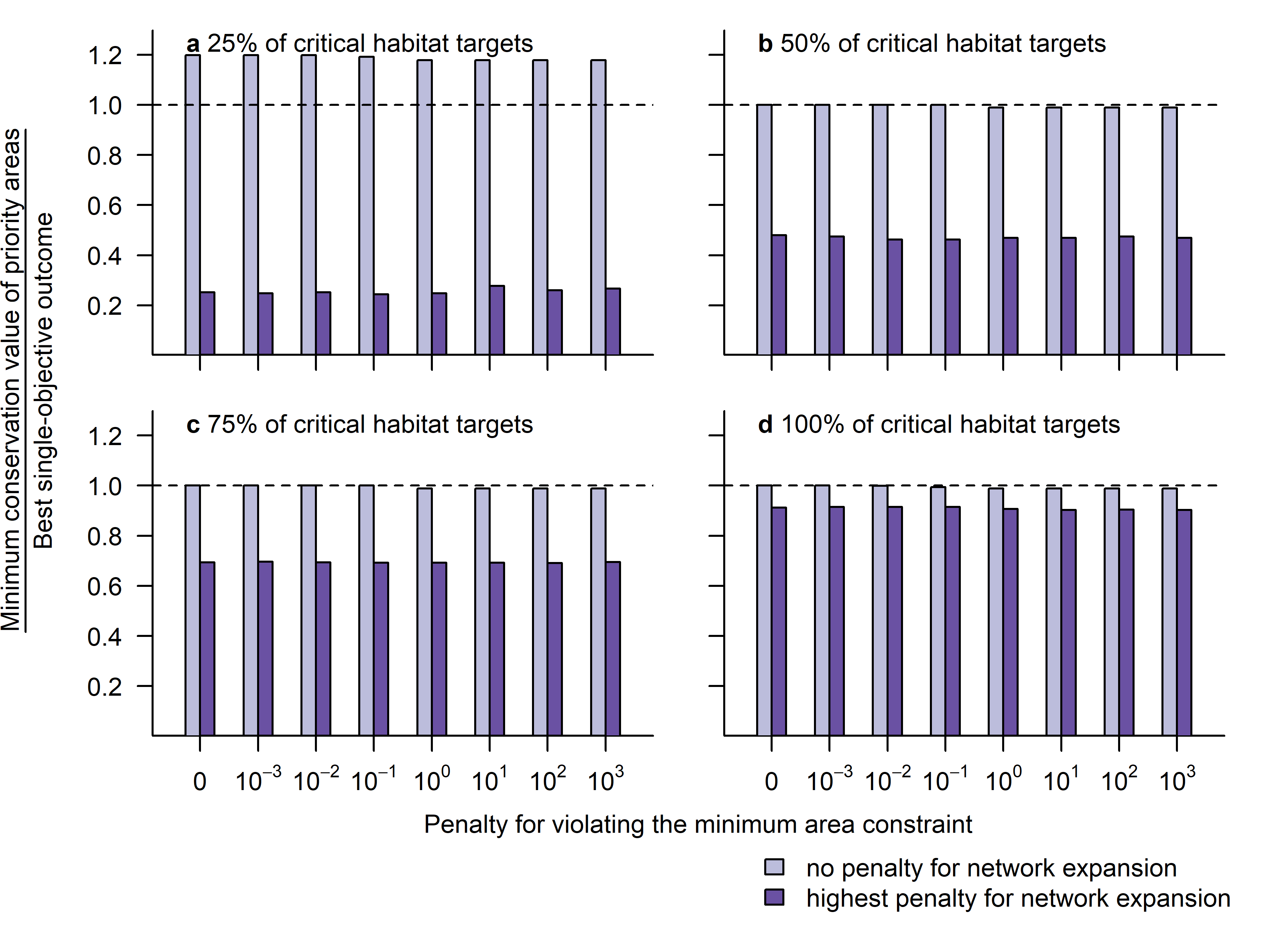


Fig. S13. How close we could get to the best possible outcome when simultaneously prioritizing for all our conservation objectives in the Protect Habitat scenario was not strongly affected by the penalty for violating a minimum area constraint. Each figure depicts the minimum conservation value across the set of conservation objectives, as a proportion of the best single-objective outcome (the outcome when prioritizing to maximize each individual objective), for prioritizations using a range of penalties for expanding the protected areas network and penalties for violation of a minimum area constraint. Values >1 indicate that the minimum conservation value achieved across the set of six conservation objectives was higher in the multi-objective prioritization than when each objective was prioritized separately; this was possible when the area prioritized for protection using our multi-objective programming problem exceeded the area prioritized in the single-objective context (see Fig. S11).

References

1. Chung, N. C., Miasojedow, B., Startek, M. & Gambin, A. Jaccard/Tanimoto similarity test and estimation methods for biological presence-absence data. *BMC Bioinformatics* **20,** 644; 10.1186/s12859-019-3118-5 (2019).

2. Environment and Climate Change Canada. Amended recovery strategy of the Woodland Caribou (*Rangifer tarandus caribou*), boreal population, in Canada. Species at Risk Act Recovery Strategy Series (Environment and Climate Change Canada, 2020).

3. Johnson, C. A. *et al.* Science to inform policy: linking population dynamics to habitat for a threatened species in Canada. *J. Appl. Ecol.* **57,** 1314–1327 (2020).
